# Prognostic indicators in adults hospitalized with falciparum malaria in Western Thailand

**DOI:** 10.1186/1475-2875-12-229

**Published:** 2013-07-08

**Authors:** Paul N Newton, Kasia Stepniewska, Arjen Dondorp, Kamolrat Silamut, Wirongrong Chierakul, Sanjeev Krishna, Timothy ME Davis, Yupin Suputtamongkol, Brian Angus, Sasithon Pukrittayakamee, Ronnatrai Ruangveerayuth, Josh Hanson, Nicholas PJ Day, Nicholas J White

**Affiliations:** 1 Lao-Oxford-Mahosot Hospital-Wellcome Trust Research Unit, Microbiology Laboratory, Mahosot Hospital, Vientiane, Lao PDR; 2 Faculty of Tropical Medicine, Mahidol University, Bangkok, Thailand; 3 Centre for Tropical Medicine, Churchill Hospital, Nuffield Department of Medicine, University of Oxford, Oxford OX3 7LJ, UK; 4 Division of Clinical Sciences, St George’s Hospital, University of London, London, UK; 5 School of Medicine & Pharmacology, University of Western Australia, Freemantle, Australia; 6 Department of Medicine, Siriraj Hospital, Bangkok, Thailand; 7 Mae Sot Hospital, Mae Sot, Tak Province, Thailand; 8 Menzies School of Health Research, Darwin, Australia

**Keywords:** Malaria, Mortality, Thailand, *Plasmodium falciparum*, Prognosis

## Abstract

**Background:**

Severe malaria remains a major cause of death and morbidity amongst adults in the Asiatic tropics.

**Methods:**

A retrospective analysis of the clinical and laboratory data of 988 adult patients, hospitalized with *Plasmodium falciparum* malaria and prospectively recruited to malaria studies in western Thailand between 1986 and 2002, was performed to assess the factors associated with a fatal outcome. Different severity scores and classifications for defining severe malaria were compared and, using multiple logistic regression, simple models for predicting mortality developed.

**Results:**

The proportion of patients fulfilling the WHO 2000 definition of severe malaria was 78.1%, and their mortality was 10%. Mortality in patients given parenteral artesunate or artemether (16/317, 5%) was lower than in those given parenteral quinine (59/442, 13%) (P < 0.001). Models using parameter sets based on WHO 1990, 2000 and Adapted AQ criteria plus blood smear parasite-stage assessment gave the best mortality prediction. A malaria prognostic index (MPI), derived from the dataset using five clinical or laboratory variables gave similar prognostic accuracy.

**Conclusions:**

The mortality of severe malaria in adults has fallen and the switch from quinine to artesunate has probably been an important contributor. Prognostic indices based on WHO 2000 definitions, and other simpler indices based on fewer variables, provide clinically useful predictions of outcome in Asian adults with severe malaria.

## Background

*Plasmodium falciparum* malaria still kills ~0.7 million people each year. The majority of those who die are children in sub-Saharan Africa, but ~43,000 patients still die each year in the Asia-Pacific [1]. Admission clinical features that predict death have been defined in African children [2-6]. For Asian adults the relationships between mortality and a wide range of individual variables have been tested, including parasitaemia and parasite-stage distribution and intraleucocytic malaria pigment on blood smears, renal failure, hypoglycaemia, cerebral malaria, acidosis and older age [7-14] (Additional file 1, Tables 1 and 2). However, there have been few assessments of which combinations of prognostic factors are the best predictors of mortality. Although a single definition may not be appropriate everywhere, it is important to attempt to define severe malaria to aid clinicians in recognizing those at risk of death and in need of parenteral therapy. This will inform decisions on whether transfer to a higher level of care, if available, is appropriate, facilitate comparison between datasets, longitudinal epidemiological surveillance, and robust case definitions in evaluation of interventions [11,15].

**Table 1:** Clinical variables included in the ten sets, used in the logistic regression analyses

**Variable**	**Bed side**	**Bedside and simple lab**	**WHO 1990**	**WHO 2000**	**Adapted AQ**	**Adapted AQ plus late stages**	**BCAM**	**RCAM**	**MSA**	**MPI**
Seizures before admission	X	X	X	X	-	-	-	-	-	-
Clinical anaemia	X	X	-	-	-	-	-	-	-	-
Clinical jaundice	X	X	X	X	-	-	-	-	-	-
Bleeding	-	-	X	X	-	-	-	-	-	-
Blackwater fever	-	-	X	X	-	-	-	-	-	-
Temperature	X	X	X	-	-	-	-	-	-	-
Pulse	X	X	-	-	-	-	-	-	-	-
Respiratory rate	X	X	-	X	-	-	-	X	-	-
Respiratory distress, requiring mechanical ventilation	-	-	-	-	-	-	-	-	X	-
Liver palpable	X	X	-	-	-	-	-	-	-	-
GCS Total	X	X	X	X	X	X	X	X	X	X
Pulmonary oedema	-	-	X	X	-	-	-	-	-	-

X = included, - = not included.

BCAM score uses serum bicarbonate as a marker of acidosis with cut-off values of ≥24 mmol/L (score = 0) for normal, ≥15- < 24 mmol/L (score = 1) for deranged, and <15 mmol/L (score = 2) for very deranged along with thresholds for coma of GCS ≤14 and GCS ≤10 [23]. The acidosis and coma scores were summed to give the BCAM score, ranging from 0–4.

RCAM score uses respiratory rate as a surrogate marker for acidosis with cut offs of <20 breaths/min (score = 0) for normal, 20–39 breaths/min (score = 1) for deranged, and ≥40 breaths/min (score = 2) for very deranged.

The MSA score was defined as sum of 1 (severe anaemia [haemoglobin, <5 g/dL]) + 2 (acute renal failure [serum creatinine, >3 mg/dL or 250 μmol/L]) + 3 (respiratory distress, requiring mechanical ventilation) + 4 (cerebral malaria [GCS <11]), in which each variable was scored as 0 or 1, depending on its absence or presence, respectively [22].

**Table 2:** Laboratory variables included in the ten sets, used in the logistic regression analyses

**Blood test variable**	**Bed side**	**Bedside and simple lab**	**WHO 1990**	**WHO 2000**	**Adapted AQ**	**Adapted AQ plus late stages**	**BCAM**	**RCAM**	**MSA**	**MPI**
Admission parasitaemia	-	X	X	X	X	X	-	-	-	X
Pigmented stages	-	X	-	-	-	X	-	-	-	X
Modal Stage	-	X	-	-	-	-	-	-	-	-
Haematocrit	-	X	X	X	X	X	-	-	-	-
Haemoglobin	-	-	-	-	-	-	-	-	X	-
White cell count	-	-	-	-	-	-	-	-	-	-
Potassium ^S^	-	-	-	-	-	-	-	-	-	-
Creatinine ^S^	-	-	X	X	X	X	-	-	X	-
Urea ^S^	-	-	-	-	-	-	-	-	-	-
Urea:creatinine ratio ^S^	-	-	-	-	-	-	-	-	-	-
Total bilirubin^S^	-	-	X	-	X	X	-	-	-	X
Direct bilirubin ^S^	-	-	-	-	-	-	-	-	-	-
Alkaline phosphatase ^S^	-	-	-	-	-	-	-	-	-	-
AST ^S^	-	-	-	-	-	-	-	-	-	-
ALT ^S^	-	-	-	-	-	-	-	-	-	-
Albumin ^S^	-	-	-	-	-	-	-	-	-	-
Bicarbonate ^S^	-	-	X	X	X	X	X	-	-	-
Glucose ^P^	-	-	X	X	X	X	-	-	-	-
Lactate ^P^	-	-	-	X	X	X	-	-	-	X

X = included, - = not included. ^S^ = serum, ^P^ = plasma.

Several definitions, classifications and severity scores have been proposed. The World Health Organization (WHO) has produced three guidelines which include definitions of severe malaria (Additional file 1, Tables 1 and 2) [16-18]. A slightly stricter definition of severe malaria (‘AQ’) was developed for a clinical trial in Vietnam [19] and adapted (with the addition of plasma lactate and serum bicarbonate measurements) for a trial in Thailand [20] and the subsequent multicentre SEAQUAMAT trial which enrolled 1461 Asian patients [21]. The malaria severity assessment score (MSA), based on haemoglobin, serum creatinine, requirement for mechanical ventilation and Glasgow coma score (GCS) was developed in central India [22].

Recently, in order to simplify and, therefore, broaden usage, the coma acidosis malaria (CAM) score was developed [23], based on data from Asian adults with severe malaria [21]. A score of <2/5, when tested with data from different studies in Vietnamese and Bangladeshi adults, had a positive predictive value (PPV) for survival of 94-95%, suggesting that these patients could be cared for without admission to an intensive care unit (ICU).

The clinical and laboratory features of adults with falciparum malaria recruited prospectively to hospital-based studies on the western border of Thailand between 1986–2002, conducted by the Mahidol University-Oxford Tropical Medicine Research Unit and colleagues, were analysed. The specificity and sensitivity of the different severe malaria definitions and scoring systems were examined and simple models to identify adults at risk of death built.

## Methods

### Study sites, years and studies

Data from 988 adult (≥15 years) patients with asexual forms of *P. falciparum* present on peripheral blood slides, admitted to hospital with malaria and then recruited to clinical research studies on the western border of Thailand between 1986 and 2002 were analysed. Patients were recruited at hospitals in Kanchanaburi (1986–1993; n = 571), Sangklaburi (1994–1995; n = 74), and Mae Sot (1995–2002; n = 343) and described in a series of papers [24]. All patients gave informed consent to participation and all studies were approved by the Ethics Committee of the Faculty of Tropical Medicine, Mahidol University and/or the Ethical and Scientific Review Subcommittee of the Ministry of Public Health, Government of Thailand.

### Clinical and laboratory assessment

As patients were recruited to a variety of studies, clinical and laboratory evaluations varied. All patients had a full history and examination performed and haematocrit and parasitaemia determined. Thick and thin blood films were stained immediately with Field’s stain and parasites counted and staged [7,8]. Admission blood samples for full blood count, serum sodium, potassium, creatinine, urea, total bilirubin, direct bilirubin, alkaline phosphatase, alanine transaminase (ALT), aspartate transaminase (AST), plasma lactate, glucose and, except in 1994, for plasma bicarbonate. Lumbar punctures were performed for the majority of patients with reduced GCS and cerebrospinal fluid cell, protein and glucose concentrations determined and Gram stains examined.

### Management

The anti-malarial treatment regimens used in clinical studies in western Thailand changed over the 18 years as new anti-malarials were introduced and were:

1. Non-artemisinin-based parenteral treatment:

Intravenous quinine dihydrochloride with or without a 20 mg salt/kg or 7 mg/kg loading dose followed by 10 mg/kg every 8 h followed by oral quinine salt 10 mg/kg every 8 h, combined, when the patient was able to take oral medication, with oral quinine alone or with oral tetracycline, doxycycline, mefloquine (alone or combined with sulphadoxine-pyrimethamine (SP)) or single dose primaquine to give a total treatment course of 7 days.

2. Non-artemisinin-based oral treatment:

Oral quinine sulphate 10 mg salt/kg every 8 h alone or with oral tetracycline, doxycycline, mefloquine (alone or combined with SP), single dose primaquine or proguanil to give a total treatment course of 7 days.

Or oral mefloquine (alone or combined with SP) at 15 mg base/kg on the first day with or without 10 mg/kg on the next day

Or oral halofantrine (8 mg/kg at 0, 6, 12 h) with tetracycline plus or minus oral quinine

3. Artemisinin-based parenteral treatment:

Intravenous artesunate (Guilin Pharmaceutical Factory No. 2, Guangxi, People’s Republic of China); 2.4 mg/kg stat, 1.2 mg/kg at 12 h followed by 1.2 mg/kg every 24 h) or intramuscular artemether (3.2 mg/kg stat followed by 1.6 mg/kg every 24 h) for 7 days alone or with oral doxycycline, tetracycline or mefloquine (alone or combined with SP) with or without primaquine. Sixty-nine patients were treated with iv artesunate combined with iv quinine followed by the above oral regime [25].

4. Artemisinin-based oral treatment:

Oral artesunate or artemether (4 mg/kg for 3 days or 2 mg/kg for 7 days) alone or with oral doxycycline, tetracycline or mefloquine (alone or combined with SP) with or without primaquine. Eighteen patients received oral dihydroartemisinin (4 mg/kg) in replacement for one artesunate daily dose [26].

Supportive treatment was in accordance with guidelines [17,27]. Facilities for urinary catheter and nasogastric tube placement, blood transfusion and lumbar puncture were available at Sangklaburi Hospital. In addition, mechanical ventilation, peritoneal dialysis and central venous access were available at Kanchanaburi and Mae Sot.

### Statistical analysis and modelling

Six main models have been used to define severe malaria in adults, those published by WHO and the AQ, MSA and CAM scores [16-23] (Additional file 1). The accuracy of the APACHE II score [28] could not be assessed as not all necessary laboratory variables were measured, and the definition of WHO 1986 was not evaluated [16]. Data were not collected specifically for this analysis and in order to examine the sensitivity and specificity of WHO 1990 and 2000 definitions [17,18], minor adaptations were made to allow assessment (Additional file 1, Tables 1 and 2). Where the WHO 2000 definition [18] does not include quantitative cut offs, the WHO 1990 [17] cut offs have been used. As blood pH, and hence base deficit, data were not available, calculation of the CAM score was not possible and so the modified CAM scores, BCAM and RCAM were evaluated, using serum bicarbonate and respiratory rate, respectively, as surrogates of acidosis (Tables 1 and 2).

Statistical analyses were performed using Stata (v11.0; Stata Corporation, USA). All univariate comparisons between survivors and patients who died were performed using logistic regression and adjusted for study site. Ten sets of clinical and laboratory assay variables (Tables 1 and 2) were used to construct diagnostic rules to predict death. Logistic regression analysis with a stepwise forward variable selection procedure was employed to find independent predictors of death at P < 0.055, and P ≥ 0.055 for entry and removal, respectively. Fractional polynomials [29] were used to test for non-linear relationships between outcome variable and continuous covariates. All models were adjusted for artemisinin-based combination therapy (ACT) and study site. The identified model was rerun on the maximum available sample size and each of the variables not in the model were tested for inclusion using the Wald test. The predictive utility of each final model was assessed by receiver operating characteristic (ROC) curve analysis.

### Malaria prognostic index

Variables selected into any final logistic regression model, based on published criteria (see above), were used to define the malaria prognostic index (MPI). Since for all logistic models (above), inclusion of study site as an independent variable did not improve the model nor change the co-efficients for other covariates significantly, it was not included in the development of the MPI. Each variable was categorized into four groups using rounded quartiles or commonly used cut offs (as for GCS). Univariate logistic models (with sets of corresponding binary variables) were fitted and categories with similar (P > 0.05) odds ratios (OR) were grouped together. Co-efficients of the final multivariate model were rounded to the nearest integer and used to calculate for each patient a linear combination of variables (i. e, sum of variables multiplied by the rounded co-efficients) – the MPI. The MPI was calculated for each patient and the ROC analysis used to evaluate its prognostic utility.

The predictive value of the MPI was further evaluated using cross validation [30] on a subset of data with no missing values for variables chosen to define MPI. Each observation in this subset was sequentially removed, logistic regression with stepwise variable selection was performed on the remaining n-1 observations using all categorized variables, and the final model was used to calculate sensitivity, specificity on n-1 observations and classification results for the one excluded observation. Co-efficients were rounded to integers and cut offs for the linear predictor between two and six were used. For each cut off, classification results for observations excluded from the subsequent models were used to calculate jacknife estimates of sensitivity and specificity.

## Results

### Clinical presentation and outcome

Of 988 hospitalized patients enrolled (Tables 3, 4, 5, 6, 7 and 8), two thirds were men and ages ranged from 15 to 74 years. The percentages with severe malaria, as defined by the WHO 1990, WHO 2000, adapted AQ, BCAM, RCAM and MSA scores [17,18,20-22], were widely spread at 61.2, 78.1, 41.7, 24.8, 22.7 and 5.6%, respectively (see Additional files 2, 3, and 4). The overall mortality was 7.8% (77/988). Using the WHO 1990, 2000 and adapted AQ definitions of severe malaria [17-20], the mortalities were 12.4%, 10% and 18.7%, in these groups, respectively.

**Table 3:** Admission clinical history details for the three study sites

**Variable**	**All patients**		**Kanchanaburi**		**Sangklaburi**		**Mae Sot**	
	**N**	**Number (%)**	**N**		**N**		**N**	
No. male	982	654 (67)	569	371 (65)	74	42 (57)	339	241 (71)
Age/years	979	27 (15–74)	564	26 (15–72)	73	25 (15–57)	342	28 (15–74)
Body weight/kg	929	51 (26–165)	544	51 (32–80)	70	50 (26–89)	315	51 (32–165)
Height/cm	441	160 (96–180)	199	160 (96–78)	2	154 (145–163)	240	160 (143–180)
BMI kg/m^a^	440	33.5 (32.8-34.2)	198	33.0 (32.3-33.8)	2	42.1	240	33.8 (32.7-34.8)
Prior malaria	780	445/335 (57)	464	256/208 (55)	52	26/26 (100)	264	163/101 (62)
Prior malarial drug	988	268/720 (27)	571	233/338 (41)	74	6/68 (8)	343	29/314 (9)
No. days ill	956	4 (1–31)	557	4 (1–31)	68	3 (1–30)	331	3 (1–21)
Females pregnant	328	76/252 (23)	198	70/128 (35)	32	5/27 (16)	98	1/97 (1)
Headache	921	843/78 (92)	520	484/36 (93)	72	66/6 (92)	329	293/36 (89)
Rigors	875	454/421 (52)	491	309/182 (63)	60	15/45 (25)	324	130/194 (40)
Vomiting	906	5 17/389 (57)	511	296/215 (58)	69	37/32 (54)	326	184/142 (56)
Diarrhoea	850	160/690 (19)	461	90/371 (24)	65	8/57 (12)	324	62/262 (19)
Abdominal pain	867	223/644 (26)	478	129/349 (27)	65	7/58 (11)	324	87/237 (27)
Cough	851	143/708 (17)	470	99/371 (21)	55	6/49 (11)	326	38/288 (12)
Seizures	836	28/808 (3)	456	9/447 (2)	60	1/59 (2)	320	18/302 (6)

Number with/without symptom or sign or median (range) except ^a^ mean (95% CI) and ^b^ geometric mean (95% CI). Percentages shown in brackets. Location of hospitals included: Kanchanaburi (14.06 ^0^ N, 99.50 ^0^E), Sangklaburi 15.16 ^0^ N 98.56 ^0^E), Mae Sot (16.71 ^0^ N 98.57 ^0^E).

**Table 4:** Admission clinical examination details for the three study sites

**Variable**	**All patients**		**Kanchanaburi**		**Sangklaburi**		**Mae Sot**	
	**N**	**Number (%)**	**N**		**N**		**N**	
Dehydration	781	428/353 (55)	424	182/242 (43)	41	13/28 (32)	316	233/83 (74)
Anaemia	932	274/658 (29)	529	161/368 (30)	68	10/58 (15)	335	103/232 (31)
Jaundice	969	226/743 (23)	561	137/424 (24)	72	9/63 (13)	336	80/256 (24)
Temperature °C ^a^	963	38.5 (38.4-38.6)	552	38.4 (38.3-38.5)	74	38.5 (38.3-38.8)	337	38.7 (38.5-38.8)
Pulse/min ^a^	927	101.0 (99.9-102.2)	519	97.4 (95.9-98.9)	73	103.1 (99.2-106.9)	335	106.2 (104.6-108.0)
BP systolic mmHg ^a^	924	108.3 (107.3- 109.3)	515	107.5 (106.2-108.9)	71	105.7 (102.2-109.3)	338	110.1 (108.3-111.8)
BP diastolic mmHg ^a^	918	65.5 (64.7-66.3)	513	64.3 (63.3-65.4)	71	68.8 (66.6-71.0)	334	66.7 (65.3-68.0)
Respiratory rate/min ^b^	909	24.3 (23.8-24.7)	508	22.8 (22.2-23.3)	70	28.1 (26.6-29.8)	331	25.9 (25.2-26.6)
Abdominal tenderness	801	124/677 (15)	413	61/352 (15)	73	8/65 (11)	315	55/260 (17)
Liver palpable	870	338/532 (39)	500	183/317 (37)	68	7/61 (10)	302	148/154 (49)
Spleen palpable	885	190/695 (22)	506	94/412 (19)	71	0/71 (0)	308	96/212 (31)
Cranial nerve abnormalities	515	35/480 (7)	230	23/207 (10)	15	2/13 (13)	270	10/260 (4)
GCS Total	944	15 (3–15)	529	15 (3–15)	73	15 (3–15)	342	15 (3–15)
GCS Eyes	941	4 (1–4)	526	4 (1–4)	73	4 (1–4)	342	4 (1–4)
GCS Verbal	939	5 (1–5)	526	5 (1–5)	73	5 (1–5)	342	5 (1–5)
GCS Motor	941	6 (1–6)	526	6 (1–6)	73	6 (1–6)	342	6 (1–6)
Fundal haemorrhages	443	11/432 (3)	202	6/196 (3)	10	1/9 (10)	231	4/227 (2)

Number with/without or median (range) except ^a^ mean (95% CI) and ^b^ geometric mean (95% CI).

**Table 5:** Admission haematology laboratory details for the three study sites

**Variable**	**All patients**		**Kanchanaburi**		**Sangklaburi**		**Mae Sot**	
	**N**	**Number (%)**	**N**		**N**		**N**	
Parasitaemia /μL^b,c^	953	72,694 (64.402-82,054)	540	53,766 (45,785-63,138)	74	71,793 (51,736-99,625)	339	117,827 (95,989-144,633)
Rings %	874	97 (1–100)	486	97 (6–100)	66	98 (42–100)	322	95 (1–100)
Trophozoites %	874	3 (0–99)	486	2.5 (0–94)	66	2.0 (0–58)	323	4.0 (0–99)
Schizonts %	876	0 (0–25)	486	0 (0–12)	67	0 (0–6)	320	0 (0–25)
Pigmented stages > 10^4^/L	868	274/594 (32)	482	121/361 (25)	66	13/53 (20)	322	140/180 (43)
Trophozoites and schizonts%	874	4 (0–99)	486	3 (0–94)	66	2 (0–58)	321	5 (0–99)
Modal Stage %	833	55 (19–99)	446	56 (19–99)	66	70 (30–96)	339	53 (23–97)
Haematocrit %	962	37 (6–56)	549	37 (8–56)	74	38 (22–50)	285	38 (6–54)
Haemoglobin g/dL ^a^	761	11.3 (11.1-11.5)	476	10.9 (10.7-11.1)	0	-	314	12.0 (11.6-12.3)
White cells x 10^9^/L	889	6.8 (1.5-67.0)	519	6.6 (1.8-67)	56	5.7 (1.5-25)	292	7.1 (1.9-54)
Neutrophils %	863	75 (12–97)	507	73 (31–97)	64	74 (12–96)	281	77 (41-96)
Platelets x10^9^/L	527	74 (0.5-404)	246	105 (16–384)	0	-	329	45 (1–404)

^c^ number of asexual parasites/1000 erythrocytes on thin film x haematocrit% × 125.6 or number of asexual parasites/200 white cells on thick film assuming white cell count 8 × 10^9^/L.

**Table 6:** Admission biochemistry laboratory variables for the three study sites

**Variable**	**All patients**		**Kanchanaburi**		**Sangklaburi**		**Mae Sot**	
	**N**	**value**	**N**	**value**	**N**	**value**	**N**	**value**
Sodium mmol/L^, d S^	764	135.4 (93.8-155.0)	419	137.8 (94–155)	16	136.5 (130–141)	328	133 (111–146)
Potassium mmol/L^, e S^	761	3.81 (1.2-7.51)	417	3.8 (1.2-7.5)	16	3.70 (3.3-4.3)	331	3.9 (1.8-7.1)
Creatinine μmol/L ^f S^	880	105.6 (35.2-1056)	475	114 (35–1056)	74	97 (66–202)	334	106 (39–836)
Urea mmol/L ^g S^	892	19.5 (3.5-226.5)	484	6.5 (1.5-56)	74	6.7 (2.5-18.7)	334	7.7 (1.3-81)
Urea mmol/L:creatinine μmol/L ratio	879	0.070 (0.005-0.256)	474	0.060 (0.005-0.244)	74	0.067 (0.022-0.014)	331	0.078 (0.021-0.026)
Total bilirubin μmol/L ^b,h, S^	859	28.9 (26.8 - 31.0)	456	23.6 (21.1-26.4)	73	29.4 (24.5-35.2)	330	38.0 (34.6-41.8)
Direct bilirubin μmol/L ^I, S^	847	8.5 (0.3-488.6)	451	8.5 (0.3-487)	72	6.1 (1.5-152)	324	9.9 (1.0-389)
Alkaline phosphatase IU/L ^b,j, S^	844	43.2 (41.1-45.3)	442	31.5 (29.8-33.3)	73	34.9 (31.9-38.2)	329	69.2 (64.2-74.6)
AST IU/L ^k, S^	858	41 (0.4-1795)	455	33 (5–210)	73	43 (0.4-183)	330	52 (10–1795)
ALT IU/L ^b,l, S^	856	20.7 (19. 5–22.0)	456	19.2 (17.6-21.1)	73	24.5 (21.3-28.2)	327	22.2 (20.4-24.1)
Albumin g/L ^a,m, S^	862	33.7 (33.2-34.2)	459	31.9 (31.2-32.7)	73	38.6 (37.0-40.1)	330	35.0 (34.3-35.7)
Calcium (uncorrected) mmol/L ^a,n, S^	397	2.06 (1.47-2.56)	0	-	73	2.12 (2.09-2.15)	324	2.05 (2.04-2.07)
Phosphate mmol/L^b,o,S^	291	0.82 (0.32-2.65)	0	-	73	1.19 (0.32-1.87)	218	0.81 (0.32-2.65)
Bicarbonate mmol/L ^a,p, S^	687	20.5 (20.2-20.8)	348	21.0 (20.5-21.4)	16	20.4 (18.9-22.0)	323	20.0 (19.4-20.5)
Chloride mmol/L ^a,q, S^	752	102.2 (101.7-102.7)	407	103.7 (103.0-104.3)	16	103.3 (101.2-105.3)	329	100.2 (99.6-100.8)
Glucose mmol/L ^r, P^	846	6.4 (0.7-37. 5)	447	6.2 (1.5-38)	70	6.2 (3.5-25)	329	7.3 (0.7-31.1)
Lactate mmol/L ^s, P^	805	2.7 (0.3-27.6)	414	2.7 (0.4-28)	74	2.2 (0.9-16)	317	2.8 (0.3-22)

Number with/without or median (range) except ^a^ mean (95% CI) and ^b^ geometric mean (95% CI).

^d^ normal range 135–145 mmol/L; ^e^ normal range 3.5-5.5 mmol/L; ^f^ normal range 70–150 μmol/L; ^g^ normal range 2.5-6.7 mmol/L; ^h^ normal range 5.1-17 μmol/L; ^I^ normal range 1.7-5.1 mmol/L; ^j^ normal range 30–300 IU/L; ^k^ normal range 5–35 IU/L; ^l^ normal range 5–35 IU/L; ^m^ normal range 35–50 g/L; ^n^ normal range 2.2-2.6 mmol/L; ^o^ normal range 1.0-1.4 mmol/L; ^p^ normal range 21–28 mmol/L; ^q^ normal range 95–105 mmol/L; ^r^ normal range 3.5-5.5 mmol/L; ^s^ normal <4 mmol/L.

**Table 7:** Admission clinical variables for those who died and survived

**Variable**	**All patients**			**Survived**			**Died**			**P**
	**N**		**%**	**N**		**%**	**N**		**%**	
No. male	982	654	67	906	598	66	76	56	74	0.197
Age/years	979	27 (15–74)	-	905	27 (15–74)	-	74	26 (15–67)	-	0.410
Body weight/kg	929	51 (26–165)	-	861	51 (26–165)	-	68	50 (40–75)	-	0.715
Prior malaria	780	445/335	57	748	438/310	59	32	7/25	22	<0.001
Prior malarial drug	988	268/720	27	911	242/669	27	77	26/51	34	0.310
No. days ill	956	4 (1–31)	-	886	3 (1–31)	-	70	4 (1–20)	-	0.836
No. females pregnant	328	76/252	23	308	75/233	24	20	1/19	5	0.049
Headache	921	843/78	92	866	794/72	94	55	49/6	89	0.511
Rigors	875	454/421	52	821	428/393	52	54	26/28	48	0.522
Vomiting	906	517/389	57	855	484/371	56	51	33/18	65	0.268
Diarrheoa	850	160/690	19	805	152/653	19	45	8/37	18	0.805
Abdominal pain	867	223/644	26	823	215/608	26	44	8/36	18	0.200
Cough	851	143/708	17	806	136/670	17	45	7/38	16	0.897
Seizures	836	28/808	3	786	18/768	2	50	10/40	20	<0.001
Dehydration	781	428/353	55	734	395/339	54	47	33/14	70	0.063
Anaemia	932	274/658	29	862	236/626	27	70	38/32	54	<0.001
Jaundice	969	226/743	23	892	175/717	20	77	51/26	66	<0.001
Temperature ^o^ C ^a^	963	38.5 (38.4 - 38.6)	-	892	38.6 (38.5-38.6)	-	71	38.0 (37.7-38.3)	-	<0.001
Pulse/min ^a^	927	101.0 (99.9-102.2)	-	856	100.1 (99.0-101.2)	-	71	113.3 (108.6–117.9)	-	<0.001
BP systolic mmHg ^a^	924	108.3 (107.3-109.3)	-	851	107.9 (106.9-108.9)	-	73	113.5 (108.4– 118.5)	-	0.004
BP diastolic mmHg ^a^	918	65.5 (64.7-66.3)	-	846	65.4 (64.6-66.2)	-	72	66.8 (63.6-70.0)	-	0.247
Respiratory rate/min ^b^	909	24.3 (23.8-24.7)	-	837	23.9 (23.5-24.3)	-	71	29.2 (26.9 - 31.6)	-	<0.001
Abdominal tenderness	801	124/677	15	743	116/627	16	58	8/50	14	0.689
Liver palpable	870	338/532	39	800	299/501	38	70	39/31	56	0.005
Spleen palpable	885	190/695	22	818	172/646	21	67	18/49	27	0.308
Cranial nerve abnormalities	515	35/480	7	467	20/447	4	48	15/33	31	<0.001
GCS Total	944	15 (3–15)	-	882	15 (3–15)	-	62	9 (3–15)	-	<0.001
GCS Eyes	941	4 (1–4)	-	881	4 (1–4)	-	60	4 (1–4)	-	<0.001
GCS Verbal	939	5 (1–5)	-	881	5 (1–5)	-	58	1 (1–5)	-	<0.001
GCS Motor	941	6 (1–6)	-	881	6 (1–6)	-	60	5 (1–6)	-	<0.001
Fundal haemorrhages	443	11/432	3	389	4/385	1	54	7/47	13	<0.001

Number with/without or median (range) except ^a^ mean (95% CI) and ^b^ geometric mean (95% CI). Footnotes as for Table 3.

**Table 8:** Admission laboratory variables for those who died and survived

**Variable**	**All patients**		**Survived**		**Died**		**P**
	**N**	**value**	**N**	**value**	**N**	**value**	
Admission parasitaemia μL^b,c^	953	72,694 (64,402–82,054)	883	68,850 (60,837-77,917)	70	144,012 (86,460-239,876)	0.002
Rings%	874	97 (1–100)	811	98 (1–100)	63	48 (1–100)	<0.001
Trophozoites%	874	3 (0–99)	811	2 (0–99)	63	52 (0–99)	<0.001
Schizonts%	876	0 (0–25)	813	0 (0–6)	63	0 (0–25)	<0.001
Pigmented stages > 10^4^/L	868	274/594 (32%)	805	227/578 (28%)	63	47/16 (75%)	<0.001
Trophozoites and schizonts %	874	4 (0–99)	811	2 (0–99)	63	52 (0–99)	<0.001
Modal Stage %	833	55 (19–99)	779	56 (23–99)	54	47 (19–94)	0.001
Haematocrit %	962	37 (6–56)	888	37 (6–56)	74	33 (12–50)	<0.001
Haemoglobin g/dL ^a^	761	11.3 (11.1- 11.5)	704	11.4 (11.2- 11.6)	57	10.1 (9.5 -10.8)	<0.001
White cells x 10^9^/L	889	6.8 (1.5-67.0)	818	6.6 (1.5 - 67)	71	11.2 (2.4 - 63)	<0.001
Neutrophils%	863	75 (12–97)	796	75 (12–97)	67	72 (36–95)	0.101
Platelets 10^9^/L	527	74 (0.5-404)	491	75 (0.5 - 404)	36	50 (4–188)	0.001
Sodium mmol/L^, d, S^	764	135.4 (93.8-155.0)	698	135.3 (100.4-155.0)	66	136.7 (93.8–153.0)	0.618
Potassium mmol/L^, e, S^	761	3.81 (1.2-7.51)	696	3.80 (2.0 –7.51)	65	4.50 (1.2-7.1)	<0.001
Creatinine μmol/L ^f, S^	880	105.6 (35.2 - 1056)	810	105.6 (35.2 - 1056)	70	240 (46.6 - 880)	<0.001
Urea mmol/L ^g, S^	892	19.5 (3.5-226.5)	821	18.7 (3.5-138)	71	58.2 (10.7 - 227)	<0.001
Urea mmol/L:creatinine μmol/L ratio	879	0.070 (0.005-0.256)	809	0.065 (0.005-0.257)	70	0.078 (0.030-0.260)	<0.001
Total bilirubin μmol/L ^b,h^	859	28.9 (26.8 - 31.0)	797	26.4 (24.6-28.4)	62	90.6 (69.6-118.1)	<0.001
Direct bilirubin μmol/L ^i^	847	8.5 (0.3-488.6)	787	8.3 (0.3 - 489)	60	40.3 (1.4 - 389)	<0.001
Alkaline phosphatase IU/L ^b,j^	844	43.2 (41.1-45.3)	781	42.1 (40.0-44.2)	63	60.7 (50.5-72.8)	<0.001
AST IU/L ^k^	858	41.0 (0.4-1795)	796	40 (0.4 - 1795)	62	95 (22–1200)	0.001
ALT IU/L ^b,l^	856	20.7 (19. 5–22.0)	794	19.7 (18.5-20.9)	62	41.3 (33.6-50.8)	<0.001
Albumin g/L ^a,m^	862	33.7 (33.2-34.2)	798	33.9 (33.4-34.4)	64	30.6 (28.5-32.7)	0.001
Calcium (uncorrected) mmol/L ^a,n^	397	2.06 (1.47-2.56)	369	2.06 (1.52-2.56)	28	2.06 (1.47-2.40)	0.385
Phosphate mmol/L ^b,o^	291	0.82 (0.32-2.65)	272	0.81 (0.32-2.10)	19	1.23 (0.48-2.65)	<0.001
Serum bicarbonate mmol/L ^a,p^	687	20.5 (20.2-20.8)	629	21.0 (20.7-21.3)	58	14.7 (13.1-16.3)	<0.001
Serum chloride mmol/L ^a,q^	752	102.2 (101.7-102.7)	688	102.3 (101.9-102.7)	64	100.6 (98.0-103.2)	0.023
Plasma glucose mmol/L ^r^	846	6.4 (0.7-37. 5)	778	6.4 (0.7 - 38)	68	6.8 (2.2-18.5)	0.139
Plasma lactate mmol/L ^s^	805	2.7 (0.3-27.6)	741	2.5 (0.3 - 22)	64	8.5 (1.9 - 28)	<0.001

Number with/without or median (range) except ^a^ mean (95% CI) and ^b^ geometric mean (95% CI). Footnotes as for Tables 3, 5 and 6.

Mortality did not significantly differ between the three sites (9% in Kanchanaburi, 3% in Sangklaburi and 8% in Mae Sot; P = 0.20). Artemisinin derivatives were given to 7% of patients in Kanchanaburi, 77% in Sangklaburi and 83% in Mae Sot (P < 0.001), reflecting temporal changes in treatment policy and study protocols. Patients in Kanchanaburi had received prior malarial treatment more frequently than at other sites and women there were more often pregnant. Patients in Mae Sot presented with lower coma scores (69% with coma score of 15 compared to >80% at the other sites and 20% with coma score ≤11 compared to 9-10% in the other sites), higher parasitaemia, higher proportions of trophozoites on admission film and a greater likelihood of a palpable liver or spleen (Tables 3, 4, 5, and 6). However, other key variables such as haematocrit, bicarbonate and lactate were similar across all sites.

Forty-one admission variables were significantly associated (P < 0.05) with death on bivariate analysis (Tables 7 and 8). Variables previously associated with mortality that were not significantly associated with death in our cohort were patient age, the number of days of illness before admission, and plasma glucose. Although there was no apparent overall relationship between mortality and age, mortality was higher with increasing age for those treated with quinine (OR (95% CI) 1.029 (1.006-1.051) P = 0.012), but not for those treated with artemisinins (OR 0.953 (0.904-1.005) P = 0.078).

### Prognostic value of parasite staging

Staging of parasite development on peripheral blood smears provides prognostic information additional to the parasite count itself; the median percentage of ring stages amongst those who survived was 98% and amongst those who died 48% (P = 0.0001). The percentage of the modal parasite stage was significantly lower amongst those who died than in those who survived (P = 0.0002), suggesting that lower circulating parasite stage synchronicity was associated with death. Using the cut offs of Silamut and White of pigmented stages >10^4^/μL or a parasitaemia of >5 x 10^5^/μL [7], the sensitivity and specificity for predicting death were 84% and 67%, respectively.

### Therapeutic responses

The median (range) coma recovery time and time to death were 24 (1–188) h (N = 46) and 44 (2–641) h (N = 76) respectively (Table 9). Artemisinin derivative-based therapy was given to 378/958 (39%) of patients starting in 1993, and artesunate or artemether was given to 317/759 (42%) of those administered a parenteral anti-malarial. A significantly higher proportion of patients had severe disease (as defined using adapted AQ criteria) in the artemisinin derivative group (46%) than the non-artemisinin group (40%) (P < 0.001). However, the mortality in those given parenteral artesunate or artemether (16/317, 5%) was lower than those given parenteral quinine (59/442, 13%) (P < 0.001). No patient without severe disease who received oral anti-malarial treatment died. The post-admission development of complications such as oliguria, seizures or pulmonary oedema and the use of ventilation, lumbar puncture, transfusion and inotropes, were all associated with death (Table 10). Mortality decreased with time, from 9% in 1986–1992 to 7% in 1993–1998 and 6% in 1999–2002 (OR = 0.943 (0.896-0.994), P = 0.030). There was increasing use of artemisinin derivatives over the same periods (0%, 72% and 93% of patients, respectively) and the corresponding percentages of parenteral treatments that were with intravenous/intramuscular artemisinin derivative were 0%, 67% and 93%, respectively. After adjusting for treatment type and study site, there was no trend in mortality over time (OR = 0.961 (0.847-1.091), P = 0.538).

**Table 9:** **Anti-malarial dr**u**g treatment and outcome**

**Variable**	**All**		**Alive**		**Died**	
	**+/−**	**%**	**+/−**	**%**	**+/−**	**%**
Non-artemisinin/artemisinin derivative therapy	580/378	60.5	521/362	59.0	59/16	78.7
Parenteral quinine/artemisinin derivative. Patients given both parenteral quinine and artemisinin derivatives excluded	430/276	60.9	371/261	58.7	59/15	79.7
Oral quinine/artemisinin derivative and no parenteral anti-malarial	93/60	60.8	93/60	60.8	0/0	-
Number given/not given antibiotic ^a^	124/818	13.2	94/778	10.8	30/40	42.9
No. given/not given inotrope	65/872	6.9	13/856	1.5	52/16	76.5
No. given/not given anti-epileptic drug	97/843	10.3	59/812	6.8	38/31	55.1
No. given/not given nasogastric tube	65/859	7.0	36/828	4.2	29/31	48.3
No. given/not given urinary catheter	207/728	22.1	142/725	16.4	65/3	95.6
No. given/not given blood transfusion	160/828	16.2	132/779	14.5	28/49	36.4
No. given/not given dialysis	31/898	3.3	7/854	0.8	24/44	77.4
No. given/not given ventilation	85/845	9.1	25/839	2.9	60/6	90.9
No. given/not given central venous access line	99/840	10.5	45/825	5.2	54/15	78.3
No. given/not given lumbar puncture	90/849	9.6	56/813	6.4	34/36	48.6
No. developing/not developing pulmonary oedema	14/958	1.4	7/888	0.8	7/70	9.1
No. developing/not developing oliguria	94/843	10.0	43/826	5.0	51/17	75.0
No. developing/not developing seizures	39/909	4.1	20/852	2.3	19/57	25.0
No. of nights in hospital^b^	4 (1–34)	-	4 (1–34)	-	2 (1–27)	-
Coma Recovery Time/h^b^	24 (1–188)	-	-	-	-	-
Time to death/h^b^	-	-	-	-	44 (2–641)	-

^a^ excluding doxycycline and tetracycline given for malaria. ^b^ Median (range). All comparisons between alive and dead were statistically significant (P < 0.001, adjusted for study site).

**Table 10:** Summary of multiple logistic regression models for outcome for Bedside and WHO models

**Covariate**	**OR**	**95% CI**		**P value**	**AUROCC 95% CI**
**Bedside**					0.93 (0.90-0.96)
Anaemia	2.510	1.173	5.373	0.018	
Jaundice	3.235	1.534	6.823	0.002	
Temperature ^0^C	0.673	0.490	0.926	0.015	
Pulse/min	1.031	1.007	1.056	0.010	
Respiratory rate/min	1.061	1.010	1.115	0.018	
GCS	0.717	0.654	0.785	<0.001	
Treatment with ACT	0.387	0.134	1.118	0.080	
**Bedside + Simple Lab**					0.96 (0.93 -0.98)
Temperature ^0^C	0.579	0.405	0.829	0.003	
Respiratory rate/min	1.081	1.027	1.139	0.003	
Log_10_ parasitaemia	8.606	3.525	21.011	<0.001	
GCS	0.663	0.589	0.745	<0.001	
% Trophozoites and schizonts	1.029	1.014	1.045	<0.001	
Treatment with ACT	0.253	0.078	0.825	0.023	
**WHO (1990)**					0.95 (0.90-1.00)
GCS	0.673	0.590	0.767	<0.001	
Serum creatinine μmol/L	1.005	1.001	1.009	0.007	
Serum bicarbonate mmol/L	0.844	0.763	0.934	0.001	
Serum total bilirubin μmol/L	1.006	1.001	1.010	0.011	
Log_10_ parasitaemia	1.902	1.060	3.410	0.031	
Treatment with ACT	0.838	0.224	3.142	0.794	
**WHO (2000) and Adapted AQ **^a^					0.97 (0.96-0.99)
GCS	0.673	0.582	0.778	<0.001	
Serum bicarbonate mmol/L	0.856	0.747	0.980	0.024	
Serum lactate mmol/L	1.197	1.052	1.362	0.006	
Log_10_ parasitaemia	2.460	1.168	5.182	0.018	
Serum creatinine μmol/L	1.006	1.002	1.010	0.003	
Treatment with ACT	1.000	0.220	4.555	1.000	

All estimates are adjusted for treatment with ACT and study site. ^a^ the same variables were identified from the WHO (2000) and Adapted AQ models.

### Relationships between clinical syndromes

Of 111 patients with cerebral malaria (GCS < 11), 97 had evaluable data. Of these, 68 (70%) had plasma lactate >4 mmol/L and/or serum bicarbonate <15 mmol/L and/ or serum creatinine >264 μmol/L, and 36 (53%) of these died compared with three (10%) among 29 cerebral malaria patients without these abnormalities (P = 0.001). Among cerebral malaria patients, mortality did not significantly differ between those with and without jaundice (total serum bilirubin >50 μmol/L): 42% (23/55) *versus* 34% (15/44) (P = 0.547). Mortality was higher in patients with renal impairment; 82% (18/22) in those with a serum creatinine >264 μmol/L compared with 28% (23/83) in those with lower levels (P < 0.001).

### Multiple logistic regression analysis of variables associated with death

All final models were adjusted for treatment with ACT and study site. No interactions between covariates and treatment were significant in any of the models and study site was not significant (P > 0.05). Tables 10 and 11 list variables included in each of the eight final models, all having a maximum of eight variables, consistent with recommendations [31].

**Table 11:** Summary of multiple logistic regression models for outcome for Adapted AQ + pigmented stages, BCAM and RCAM models

**Covariate**	**OR**	**95% CI**		**P value**	**AUROCC 95% CI**
**Adapted AQ + pigmented stages**					0.97 (0.96-0.99)
GCS	0.728	0.641	0.828	<0.001	
Plasma lactate mmol/L	1.341	1.190	1.510	<0.001	
Serum creatinine μmol/L	1.006	1.003	1.009	<0.001	
% Trophozoites & schizonts	1.026	1.009	1.043	0.003	
Log_10_ parasitaemia	4.064	1.506	10.969	0.006	
Treatment with ACT	0.438	0.103	1.869	0.265	
**BCAM**					0.92 (0.87-0.97)
GCS/15	0.718	0.652	0.791	<0.001	
Serum bicarbonate mmol/L	0.780	0.717	0.849	<0.001	
Treatment with ACT	0.718	0.230	2.237	0.568	
**RCAM**					0.88 (0.84-0.93)
GCS/15	0.680	0.627	0.736	<0.001	
Respiratory rate/min	1.097	1.055	1.141	<0.001	
Treatment with ACT	0.329	0.126	0.857	0.023	

All estimates are adjusted for treatment with ACT and study site.

Considering the WHO- and AQ-based models, the WHO 1990, WHO 2000, Adapted AQ and Adapted AQ + Pigmented stages, variable sets gave the best predictive power and the areas under the ROC curves (AUROCCs) were not significantly different (Tables 10 and 11, Additional files 2, 3, and 4). Models derived from bedside or bedside + simple laboratory covariates had significantly smaller AUROCCs, when compared to the Adapted AQ model using the common data set (P = 0.008 and 0.016, respectively). Several variables appeared in all four ‘best’ models and had similar effects across models, such as GCS (OR 0.67-0.73 for increase by one point), serum bicarbonate (OR 0.84-0.86 for increase of 1 mmol/L), plasma lactate (OR 1.20-1.34 for increase of 1 mmol/L), parasitaemia (OR 1.90-4.06 for ten-fold increase) and serum creatinine (OR 1.005-1.006 for increase of 1 μmol/L).

The BCAM and RCAM scores could be calculated for 662 and 873 patients, respectively, and mortality rose with increasing score for both (Table 12). Of 498 patients with a low BCAM (<2) score, three (0.6%) died (PPV (95%CI) for survival 99.4 (98.2–99.8)%), and of the 675 patients with low RCAM (<2) score, 11 (1.6%) died (PPV for survival 98.4 (97.1-99.1)%). The BCAM score AUROCC as a predictor for mortality was 0.907 (0.861-0.953) and that for the RCAM score 0.86 (0.81-0.91)(P = 0.050). A score <2 was an optimal cut-off value in this data set for both BCAM and RCAM, with sensitivity and specificity of 93.8% and 80.6% for BCAM and 81% and 81% for RCAM.

**Table 12:** Mortality among patients by BCAM and RCAM scores

**Score, Outcome**	**0**	**1**	**2**	**3**	**4**	**Total**
**BCAM**						
Died/total (%)	1/124 (0.8)	2/374 (0.5)	12/79 (15.2)	16/60 (26.7)	17/25 (68.0)	48/662 (7.3)
**RCAM**						
Died/total (%)	0/97 (0)	11/578 (1.9)	12/108 (11.1)	25/74 (33.8)	11/16 (68.8)	59/873 (6.8)

For the MSA score [22] (Additional file 1) using admission variables plus the presence or absence of mechanical ventilation during admission where such ventilation was available (all sites except Sangklaburi) for 635 patients, 516 (81.3%) had an MSA score of 0. Mortality was 2/539 (0.4%) for MSA 0–2, 10/45 (22.2%) for MSA 3–4, 5/12 (41.7%) for MSA 5–6 and 24/39 (61.5%) for MSA ≥7. If ≥5 is taken as the cut off, the PPV for survival was 97.9 (96.4-98.8)%. Among patients who had MSA and BCAM scores calculated (n = 527) the AUROCC for the MSA score in predicting death was 0.97 (0.95-0.98), which was significantly better than that for the BCAM score 0.90 (0.84-0.96) (P = 0.007). The optimal cut off for the data presented here appeared to be <3 rather than <5 as reported [22]; with a cut off of <3 the PPV was 99.6 (98.7-100.0)%, sensitivity 95.1% and specificity 90.4%.

**Figure 1:**
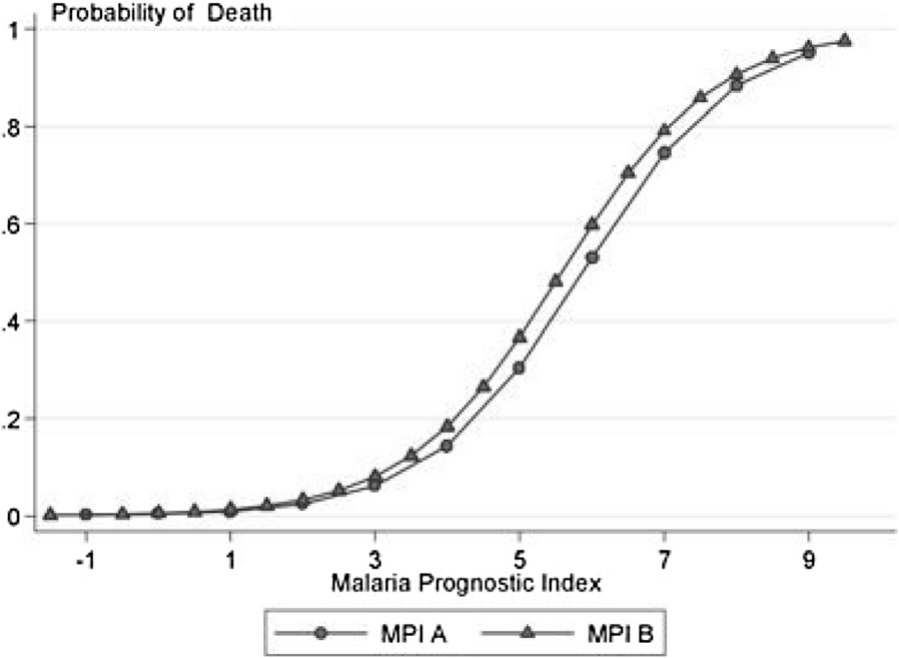
**Relationship between malaria prognostic index and mortality.** Malaria prognostic indices MPI A and MPI B are defined in Table 13.

**Figure 2:**
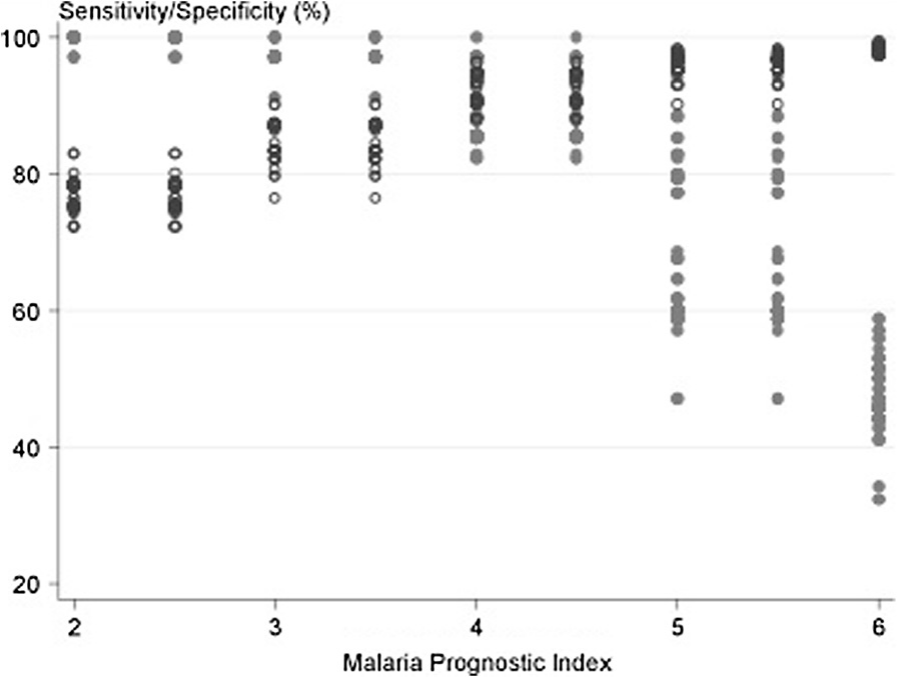
Sensitivity (filled circle) and specificity (empty circle) for different cut offs of malaria prognostic index in cross validation.

Considering all ten models, those based on the MSA score and the Adapted AQ with pigmented parasites had the best predictive power, but AUROCCs for models based on WHO criteria were not significantly lower. Simple rules for classification of severity as a MPI (Table 13, Figure 1), gave sensitivity of 100% and specificity of 82% with co-efficient of 3 rounded to the nearest integer, and sensitivity of 93% and specificity of 92% for a cut off of 4. When compared to the best performing models, based on MSA and Adaptive AQ + Pigmented stages, MPI showed equally good predictive power: AUROCC = 0.96 (0.95-0.98) for MPI rounded to the nearest integer, and 0.97 (0.96-0.99) for MPI rounded to the nearest 0.5 compared to 0.98 (0.96-0.99) for MSA and 0.97 (0.94-0.99) for AQ dataset, based on 450 patients who could be evaluated in all four models. In cross-validation, in all runs, the best sensitivity and specificity were obtained for cut offs between 3 and 4 (Figure 2). For a cut off of 3, the jacknife sensitivity and specificity were 97.1% and 87.1%; for a cut off of 3.5 they were 97.1% and 87.1%; and for a cut off of 4 they were 74% and 94%.

**Table 13:** Prognostic index for severe malaria

**N = 668 / n = 43**	**Co-efficient**		
		**MPI A**	**MPI B**
GCS <5	4.318	4	4.5
GCS 5-11	1.543	2	1.5
Parasitaemia >315,000/μL	1.238	1	1
Plasma lactate > 5 mmol/L	2.267	2	2.5
Serum bilirubin > 58 μmol/L^1^	1.191	1	1
Pigmented parasites >20%	1.673	2	1.5
Treatment with ACT	−1.280	−1	−1.5
AUROCC	0.97	0.97	0.97
Cut off ^2^		3	3
Sensitivity		100%	100%
Specificity		82%	88%
Cut off ^3^		4	3.5
Sensitivity		93%	95%
Specificity		92%	91%

MPI A = co-efficients rounded to the nearest integer with cut offs of 3.

MPI B = co-efficients rounded to the nearest 0.5 with cut offs of 3.

^1^ if not available; creatinine >132 μmol/L can be used instead.

and cut off of 3 would give sensitivity and specificity of 98%.

and 82% for MPI A and 96% and 88% for MPI B.

^2^ which gives the highest specificity for 100% sensitivity.

^3^ which gives the “optimal” sensitivity and specificity.

## Discussion

The prognosis of severe falciparum malaria has improved markedly since the introduction of parenteral artesunate [21,32]. This large series describing patients with falciparum malaria admitted to hospitals in western Thailand spanned the transition from quinine to artemisinins, and although the data were not from a randomized comparison, mortality was substantially lower in patients who received artemisinin derivatives. Despite the effect of changes in anti-malarial therapy, prognostic indices based on WHO 2000 definitions, and other simpler indices based on fewer variables, provided clinically useful predictions of outcome in Asian adults with severe malaria.

Models using variable sets based on WHO 2000 and Adapted AQ with pigmented stages gave the best prediction of mortality, and were comparable to the results based on the MSA score using a smaller sample. Very similar results were also obtained with the MPI based on the most commonly selected variables, GCS, plasma lactate, parasitaemia, serum bilirubin and percentage of pigmented parasites. This will need to be evaluated in independent series of adults with falciparum malaria in similar settings of unstable malaria transmission. This score suffers from the disadvantage, unlike the RCAM score, that determination of 4/5 variables (all except GCS) requires skilled technicians and equipment/consumables and quality assurance that are seldom available where severe malaria is common.

There are at least four limitations of this analysis: specifically, not all patients admitted to the study hospitals were recruited, recruitment criteria varied, there are missing values, and a variety of different doctors reviewed the patients with consequent variability in the nature of both clinical assessment and inpatient management. However, any such differences are likely to result in false negative, rather than false positive associations. The MSA score differs from the others scores discussed, as it is not strictly an admission predictive prognostic score, including mechanical ventilation during hospitalization. Some potential prognostic factors such as haemoglobinuria and abnormal bleeding were too infrequent to allow reliable conclusions to be drawn. Serum bilirubin and plasma lactate were not measured in the South East Asian Quinine Artesunate Malaria Trial (SEAQUAMAT) and therefore the MPI could not be calculated for this dataset [21].

This study differs from that of Hanson [23], which only included patients classified as having severe malaria and was designed to determine which subset of patients could safely be managed without ICU referral. This is reflected in the relatively low mortality in the series described here, as severe malaria was not necessarily a criterion for recruitment. The broader patient population included may well explain the inclusion of parasitaemia as predictive of death in this series and not in that of Hanson [23]. As this series includes many patients without severe disease, the specificity of variables in predicting death may be higher than in series including patients with pre-selected severe disease. However, in busy clinical practice a tool that could define unselected patients admitted to hospital as at risk of death would be valuable.

The wide variation in mortality estimates in this dataset among those with severe malaria (10-18%) and indeed the wide range of estimates of severe disease (6–60%), depending on which definition is used, illustrates the importance of definitions in comparisons between studies, in meta-analyses, and in understanding the host and geographical variability in the presentation and outcome of severe malaria. Various terms, such as ‘uncomplicated’ and ‘mild’, are used to refer to malaria that is not severe. Severe disease is also equated with ‘complicated’ malaria. To avoid confusion, terminology should be standardized with ‘severe’ malaria referring to malaria with clinical and/or laboratory features suggesting a clinically significant risk of death (e.g. >5%) despite anti-malarial treatment, and ‘uncomplicated’ as those without such prognostic features and that the terms ‘complicated’ and ‘mild’ are not used.

The present study suggests that, if laboratory tests are available, the history of the illness and the physical examination, apart from GCS and respiratory rate, are relatively unimportant in assessing prognosis in a population of malaria patients. Unlike in other series [13], increased age was not associated with death, except for those treated with quinine. However, as this series did not include children and a smaller percentage (7.7%) were >50 years old, the age range was narrower.

In this series, the mortality of cerebral malaria was increased three-fold if concurrent acidosis and/or renal failure were present and these factors are crucial in predicting death. The ease of identifying patients with cerebral malaria may have made study of other complications less of a focus and these data suggest that more research on the pathophysiology and treatment of acidosis and renal dysfunction would provide information that would improve management and outcome [12,14,32]. The use of relatively inexpensive plasma lactate portable meters may assist in the triage of patients in tropical hospitals without access to biochemical analyzers, and interventions directed at acidosis and renal impairment might reduce mortality. Although capital costs for veno-venous haemofiltration are relatively high, and intensive care support is required, running costs in comparison to peritoneal dialysis are low [33]. In areas of the Asiatic tropics with good communications but low health expenditure, regional centres for the management of severe malaria through haemofiltration may assist in lowering mortality.

## Competing interests

The authors declare that they have no competing interest.

## Authors’ contributions

PN and KS analysed the data and wrote the first draft of the manuscript. PN, AD, KS, WC, SK, TMED, YS, BA, SP, RR, JH, ND, NW looked after the patients and revised the manuscript. All authors read and approved the final manuscript.

## Supplementary Material

Additional file 1 Severe malaria definitions. Click here for file

Additional file 2 World Health Organization (1990) criteria for severe malaria and outcome. Click here for file

Additional file 3 World Health Organization (2000) criteria for severe malaria and outcome. Click here for file

Additional file 4 **Adapted ‘AQ’ criteria, from Hien *et al.***[19,20], and outcome. Click here for file
