# Supplementary material for: Prognostic indicators in adults hospitalized with falciparum malaria in Western Thailand
Source: Malar J. 2013 Jul 8;12:229. doi: 10.1186/1475-2875-12-229 (PMC3711784; doi:10.1186/1475-2875-12-229)
Supplement: Additional file 1 — Severe malaria definitions. [file 1475-2875-12-229-S1.doc]

## **Additional File-1. Severe malaria definitions.** Text in [ ] are qualifying comments taken from the text of these publications. Authors interpretations are underlined

**WHO 1990 [17]**  **WHO 2000 [18] Adapted from Hien *et al.* (1996)[19,20]**

‘Defining Criteria of severe disease’[‘in presence of asexual ‘presence of one or more of the clinical or ‘trial.. in adults with severe falciparum...

parasitemia’]^x^ laboratory features … classifies the patient as malaria…if they…had one or more of

suffering from severe disease’ ^x^ the following..’ ^x,x^

**Clinical manifestations**

1. Cerebral malaria [unrousable coma, assume GCS <9, 1. Prostration (assume=1990) 1. GCS<11

for >30 minutes after seizure. No bacterial/viral cause]

1. Severe normocytic anemia [hct < 15 % or Hb<5g/dL 2. Impaired consciousness [GCS<9] 2. Hct <20 % with parasitemia

###### with parasitemia >10,000/μL. If anaemia hypochromic >100,000/μL

and/or microcytic iron deficiency and hemogloginopathy

must be excluded]

1. Renal failure [urine output <400ml/24h with serum 3. Respiratory distress [acidotic breathing] 3. Jaundice, bilirubin >50umol/L

creatinine >265 μmol/L] (Respiratory Rate > 20/min ^1^) with parasitemia >100,000/μL

4. Pulmonary oedema or adult respiratory distress syndrome 4. Multiple convulsions (assume=1990) 4. Acute renal failure [urine<400ml/24h

5. Hypoglycaemia [whole blood glucose <2.2 mmol/L] 5. Circulatory collapse (assume=1990) with creatinine >264μmol/L]

5. Hypoglycaemia [venous glucose

6. Circulatory collapse or shock [BP systolic <70 mmHg 6. Pulmonary oedema [radiological] <2.2 mmol/L]

with cold clammy skin or core-skin temperature 6. Systolic BP <80 mmHg with

difference >10 ^0^C] cool extremities

7. Spontaneous bleeding from gums, nose, GI tract etc and 7. Abnormal bleeding (assume=1990) 7. Peripheral asexual stage parasitemia

/or substantial evidence of DIC >10 %

8. Repeated generalised convulsions [>2 in 24 hours] 8. Jaundice [clinical] 8. Peripheral venous lactate >4 mmol/L^3^

9. Acidemia/acidosis [arterial pH <7.25 or plasma 9. Hemoglobinuria (assume=1990) 9. Peripheral venous bicarbonate

bicarbonate <15 mmol/L] <15 mmol/L

10. Macroscopic hemogolbinuria [not the result of oxidant

antimalarial drugs in patients with erythrocyte enzyme defects]

**Other Manifestations** ‘which do not themselves define the **Laboratory Findings**

condition in all geographical areas and age groups’

1. Impairment of consciousness but rousable (GCS 9-14) 1. Severe anaemia (assume=1990)
2. Prostration or weakness so that patient cannot sit or walk 2. Hypoglycaemia (assume=1990)

with no obvious neurological explanation

1. Hyperparasitemia [variable definition depending on age 3. Acidosis (assume=1990)

and immunity; assume >5 %]

1. Jaundice detected clinically or serum bilirubin >50umol/L 4. Hyperlactatemia (assume >5 mmol/L ^2^)
2. Hyperpyrexia [rectal temp >40^0^C] 5. Hyperparasitemia (assume >5 %)

6. Renal impairment [assume=1990]

^1^ we chose >20 breaths/minute as abnormal; ^2^ see Krishna S, Waller DW, ter Kuile F et al. [Lactic acidosis and hypoglycaemia in children with severe malaria: pathophysiological and prognostic significance.](http://www.ncbi.nlm.nih.gov/pubmed/8154008) Trans R Soc Trop Med Hyg, **1994**; 88: 67-73.; ^3^ not included in the original definition cited in Hien *et al.* [19]

**BCAM and RCAM** [23]

**Variable Score**

0 (normal) 1 (deranged) 2 (very deranged)

GCS 15 >10 to 14 <10

Bicarbonate score >24 15 to <24 <15 mmol/L

Respiratory rate score <20 20 to <40 >40 breaths/minute

BCAM or Bicarbonate-based CAM score (0–4) is the bicarbonate score (0–2) plus the GCS (0–2)

RCAM or Respiratory rate– based CAM score (0–4) the respiratory score (0–2) plus the GCS score (0–2)

**Malaria Severity Assessment Score (MSA)** [22]

1 (severe anemia [hemoglobin level, <5 g/dL]) + 2 (acute renal failure [creatinine level, >3mg/dL]) + 3 (respiratory distress, requiring mechanical ventilation) + 4 (cerebral malaria [GCS <11]), in which each variable was scored as 0 or 1, depending on its absence or presence, respectively.
