# Supplementary material for: Prognostic indicators in adults hospitalized with falciparum malaria in Western Thailand
Source: Malar J. 2013 Jul 8;12:229. doi: 10.1186/1475-2875-12-229 (PMC3711784; doi:10.1186/1475-2875-12-229)
Supplement: Additional file 2 — World Health Organization (1990) criteria for severe malaria and outcome. [file 1475-2875-12-229-S2.doc]

**Additional File - 2. World Health Organisation (1990) criteria for severe malaria and outcome**

| Variable | All | | Alive | | Died | | P |
| --- | --- | --- | --- | --- | --- | --- | --- |
| Defining criteria | +/- | % | +/- | % | +/- | % |  |
| 1 Cerebral malaria (unrousable coma, assume GCS < 9) | 59/885 | 6.3 | 33/849 | 3.7 | 26/36 | 41.9 | <0.001 |
| 2 Severe normocytic anaemia (hct < 15 % or Hb <5 g/dL with parasitemia >10,000/μL)^a^ | 12/940 | 1.3 | 9/873 | 1.0 | 3/67 | 4.3 | 0.037 |
| 3 Renal failure (urine output <400ml/24h with serum creatinine >265 μmol/L) ^b^ | 57/823 | 6.5 | 27/783 | 3.3 | 30/40 | 42.9 | <0.001 |
| 4 Pulmonary oedema or adult respiratory distress syndrome | 8/980 | 0.8 | 3/908 | 0.3 | 5/72 | 6.5 | <0.001 |
| 5 Hypoglycaemia (whole blood glucose < 2.2 mmol/L) | 5/841 | 0.6 | 5/773 | 0.6 | 0/68 | 0 | 1.0* |
| 6 Circulatory collapse, shock (BP systolic < 70 mmHg) with cold clammy skin or core-skin temperature difference > 10 ^0^C ^c^ | 2/922 | 0.2 | 1/850 | 0.1 | 1/72 | 1.4 | 0.096 |
| 7 Spontaneous bleeding from gums, nose, GI tract etc and/or substantial laboratory evidence for DIC | 3/985 | 0.3 | 2/909 | 0.2 | 1/76 | 1.3 | 0.150 |
| 8 Repeated generalised convulsions (>2 in 24 hours) | 2/834 | 0.2 | 2/784 | 0.3 | 0/50 | 0 | 1.0* |
| 9 Acidemia/acidosis (arterial pH <7.25 or plasma bicarbonate <15 mmol/L) ^d^ | 63/624 | 9.2 | 33/596 | 5.3 | 30/28 | 51.7 | <0.001 |
| 10 Macroscopic hemoglobinuria [not the result of oxidant antimalarial drugs in patients with erythrocyte enzyme defects] | 5/983 | 0.5 | 5/906 | 0.6 | 0/77 | 0 | 1.0* |
| **Other Manifestations** | | | | | | | |
| 1 Impaired consciousness but rousable ^e^ | 123/821 | 13.0 | 99/783 | 11.0 | 24/38 | 38.7 | <0.001 |
| 2 Prostration, extreme weakness ^f^ | - | - | - | - | - | - | - |
| 2 Hyperparasitaemia [> 5%] ^g^ | 283/685 | 29.2 | 245/651 | 27.3 | 38/34 | 52.8 | <0.001 |
| 3 Jaundice (clinical jaundice or serum bilirubin >50μmol/L) | 341/644 | 34.6 | 280/628 | 30.8 | 61/16 | 79.2 | <0.001 |
| 4 Hyperpyrexia (rectal temp > 40^0^C) ^h^ | 190/773 | 19.7 | 180/712 | 20.2 | 10/61 | 14.1 | 0.209 |
|  | | | | | | | |
| One or more WHO (1990) criteria positive | 637/155 | 80.4  (70.4)# | 560/155 | 78.3  (68.8) | 77/0 | 100.0 | <0.001* |

Notes: ^a ‘^hypochromic and/or microcytic iron deficiency and hemoglobinopathy’ not included; ^b^ based on creatinine and not urine output; ^c^ core-skin temperature difference not measured, not included; ^d^ arterial pH measurements not available; ^e^ interpreted as GCS 9-14; ^f^ not recorded and excluded here; ^g^ interpreted as >5% parasitemia; ^h^  rectal temperature not measured, interpreted as 39.5 ^0^C axillary temperature.

*Fisher’s exact test used due to small numbers; #percentage calculated only in patients with all criteria evaluated
