# Supplementary material for: Prognostic indicators in adults hospitalized with falciparum malaria in Western Thailand
Source: Malar J. 2013 Jul 8;12:229. doi: 10.1186/1475-2875-12-229 (PMC3711784; doi:10.1186/1475-2875-12-229)
Supplement: Additional file 3 — World Health Organization (2000) criteria for severe malaria and outcome. [file 1475-2875-12-229-S3.doc]

**Additional File - 3. World Health Organisation** (**2000) criteria for severe malaria and outcome.**

| Variable | All | | | Alive | | | Died | | | P | |
| --- | --- | --- | --- | --- | --- | --- | --- | --- | --- | --- | --- |
|  | +/- | % | | +/- | | % | +/- | | % |  | |
| Clinical Manifestations | | | | | | | | | | | |
| 1 Prostration ^a^ | - | - | | - | | - | - | | - | - | |
| 2 Impaired consciousness (GCS<9) ^b^ | 59/885 | 6.3 | | 33/849 | | 3.7 | 26/36 | | 41.9 | <0.001 | |
| 3 Respiratory distress (acidotic breathing) ^c^  (RR > 20/min) | 612/297 | 67.3 | | 556/281 | | 66.4 | 56/16 | | 77.8 | 0.023 | |
| 4 Multiple convulsions (Repeated generalised convulsions (>2 in 24 hours)) ^d^ | 2/834 | 0.2 | | 2/784 | | 0.2 | 0/50 | | 0 | 1.0* | |
| 5 Circulatory collapse ^d^ | 2/922 | 0.2 | | 1/850 | | 0.1 | 1/72 | | 1.4 | 0.15* | |
| 6 Pulmonary edema (radiological) ^e^ | 8/980 | 0.8 | | 3/908 | | 0.3 | 5/72 | | 6.9 | <0.001 | |
| 7 Abnormal bleeding ^d^ | 3/985 | 0.3 | | 2/909 | | 0.2 | 1/76 | | 1.3 | 0.150 | |
| 8 Jaundice (clinical jaundice) | 226/743 | 23.3 | | 175/717 | | 19.6 | 51/26 | | 66.2 | <0.001 | |
| 9 Hemoglobinuria ^d^ | 5/983 | 0.5 | | 5/906 | | 0.6 | 0/77 | | 0 | 1.0* | |
| Laboratory features | | | | | | | | | | | |
| 1 Severe anemia [Severe normocytic anaemia (Hct < 15 % or Hb <5 g/dL] | 16/968 | 1.6 | | 12/895 | | 1.3 | 4/73 | | 5.1 | 0.021 | |
| 2 Hypoglycemia (whole blood glucose < 2.2 mmol/L) ^d^ | 5/841 | 0.6 | | 6/772 | | 0.6 | 0/68 | | 0 | 1.0* | |
| 3 Acidosis ^d^ | 63/624 | 9.2 | | 33/596 | | 5.2 | 30/28 | | 51.7 | <0.001 | |
| 4 Hyperlactatemia (> 5 mmol/L) | 159/646 | 19.8 | | 109/632 | | 14.7 | 50/14 | | 78.1 | <0.001 | |
| 5 Hyperparasitemia (>4 %) | 335/633 | 34.6 | | 292/604 | | 32.6 | 43/29 | | 59.7 | <0.001 | |
| 6 Renal impairment (assumed urine output <400ml/24h with serum creatinine >265 μmol/L) ^d^ | 57/823 | 6.5 | | 27/783 | | 3.3 | 30/40 | | 42.9 | <0.001 | |
|  | | | | | | | | | | | |
| **One or more WHO (2000) criteria positive** | | | 772/78 | | 90.8  (84.0)# | 695/78 | 89.9  (83.1)# | 77/0 | | 100 | 0.001* |

Notes: ^a^ not recorded and excluded here; ^b^ in Table 1 (corrected and original versions) of WHO 2000, severe malaria is indicated as ‘Impaired Consciousness’ suggesting a GCS <14/15. However, in the text S1/11, severe malaria is defined as ‘unrousable coma’ (i.e GCS <9). We have used GCS < 9 here; ^c^ to quantify respiratory distress we have chosen a cut off of greater than 20 breaths/minute; ^d^ same definition as used in WHO 1990; ^e^  as radiology was often not available in the care of these patients, a clinical diagnosis of pulmonary oedema on admission was used

*Fisher’s exact test used due to small numbers; #percentage calculated only in patients with all criteria evaluated
