# Supplementary material for: Prognostic indicators in adults hospitalized with falciparum malaria in Western Thailand
Source: Malar J. 2013 Jul 8;12:229. doi: 10.1186/1475-2875-12-229 (PMC3711784; doi:10.1186/1475-2875-12-229)
Supplement: Additional file 4 — Adapted ‘AQ’ criteria, from Hien et al.[19,20], and outcome. [file 1475-2875-12-229-S4.doc]

**Additional File - 4. Adapted ‘AQ’ criteria, from Hien *et al*. [19,20], and outcome.**

| **Variable** | **All** | | **Alive** | | **Died** | | **P** |
| --- | --- | --- | --- | --- | --- | --- | --- |
|  | +/- | % | +/- | % | +/- | % |  |
| GCS <11 | 103/841 | 10.9 | 63/819 | 7.1 | 40/22 | 64.5 | <0.001 |
| Hct <20 % with parasitemia >100,000/uL | 17/934 | 1.8 | 13/868 | 1.5 | 4/66 | 5.7 | 0.022 |
| Jaundice, bilirubin >50umol/L with parasitemia >100,000/uL | 161/676 | 19.2 | 128/651 | 16.4 | 33/25 | 56.9 | <0.001 |
| Acute renal failure (urine<400ml/24h with creatinine >264μmol/L) | 57/823 | 6.5 | 27/783 | 3.3 | 30/40 | 42.9 | <0.001 |
| Hypoglycaemia (venous glucose <2.2 mmol/L) | 5/841 | 0.6 | 5/773 | 0.6 | 0/68 | 0 | 1.0* |
| Systolic BP < 80 mmHg with cool extremities | 11/913 | 1.2 | 9/842 | 1.1 | 2/71 | 2.7 | 0.215 |
| Peripheral asexual stage parasitemia > 10 % | 135/833 | 14.3 | 106/790 | 12.2 | 29/43 | 40.0 | <0.001 |
| Peripheral venous lactate > 4 mmol/L | 218/587 | 27.1 | 161/580 | 21.7 | 57/7 | 89.1 | <0.001 |
| Peripheral venous bicarbonate < 15 mmol/L | 63/624 | 9.2 | 33/596 | 5.3 | 30/28 | 51.7 | <0.001 |
|  | | | | | | | |
| One or more criteria positive | 404/308 | 56.7  (46.0)# | 328/308 | 51.6  (42.3)# | 76/0 | 100 | <0.001* |

*Fisher’s exact test used due to small numbers; #percentage calculated only in patients with all criteria evaluated
